# Supplementary material for: The Transcriptional Regulator Prdm1 Is Essential for the Early Development of the Sensory Whisker Follicle and Is Linked to the Beta-Catenin First Dermal Signal
Source: Biomedicines. 2022 Oct 20;10(10):2647. doi: 10.3390/biomedicines10102647 (PMC9599752; doi:10.3390/biomedicines10102647)
Supplement: Supplementary file 1 [file biomedicines-10-02647-s001.zip › biomedicines-1936530-supplementary-uplated.pdf]

Supplementary Materials:

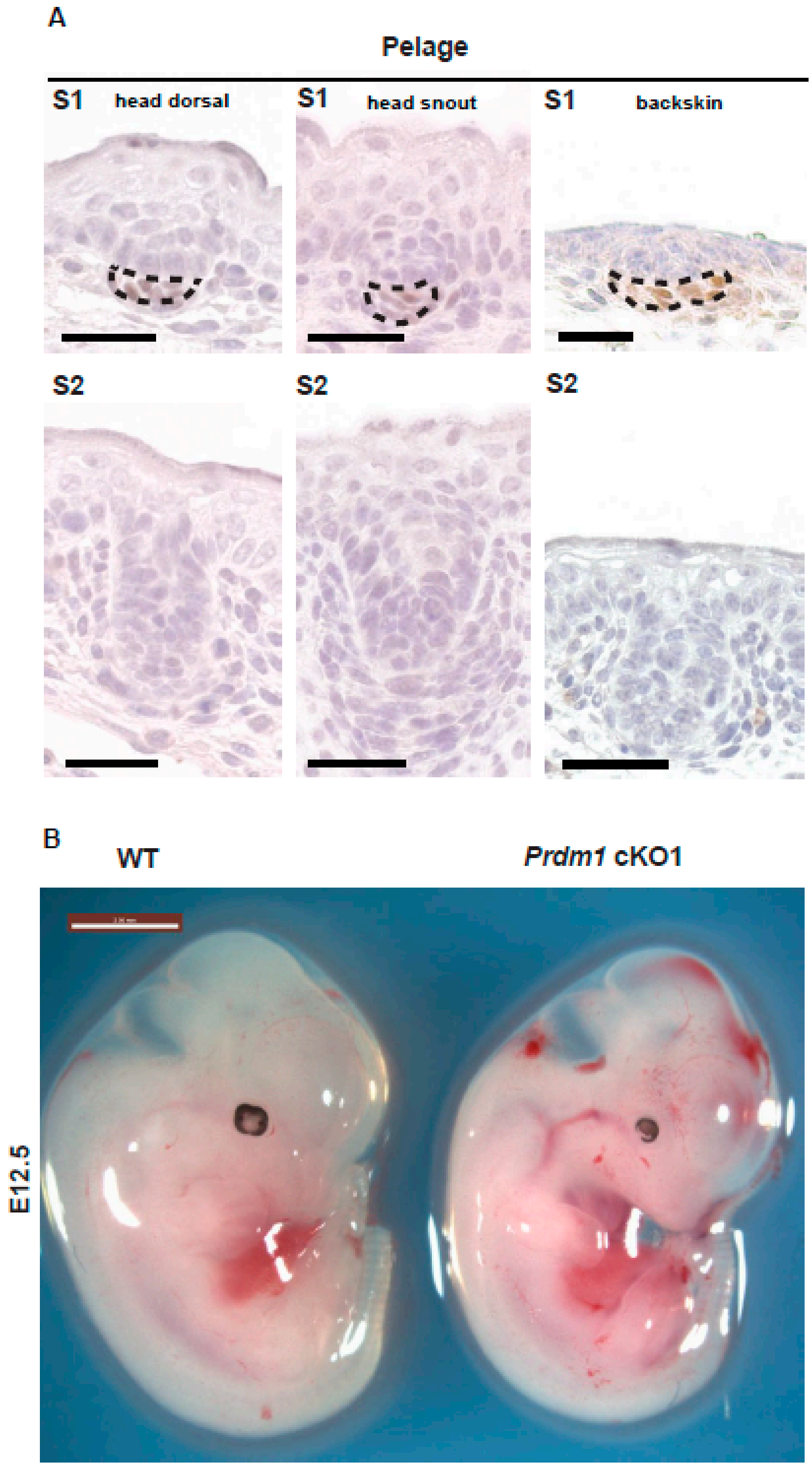

Figure S1. (A) *Prdm1* IHC on developing head/back pelage hair follicles. *Prdm1* expression in pelage hair follicle development is transient, lasts until hair germ formation (S2), and is independent of the embryonic origin of the mesenchyme. Dashed circles envelop the areas where *Prdm1* is expressed. Scale bar: 50 µm. (B) WT E12.5 embryo compared to *Prdm1* cKO1. cKO1 embryos display no whisker placodes macroscopically.

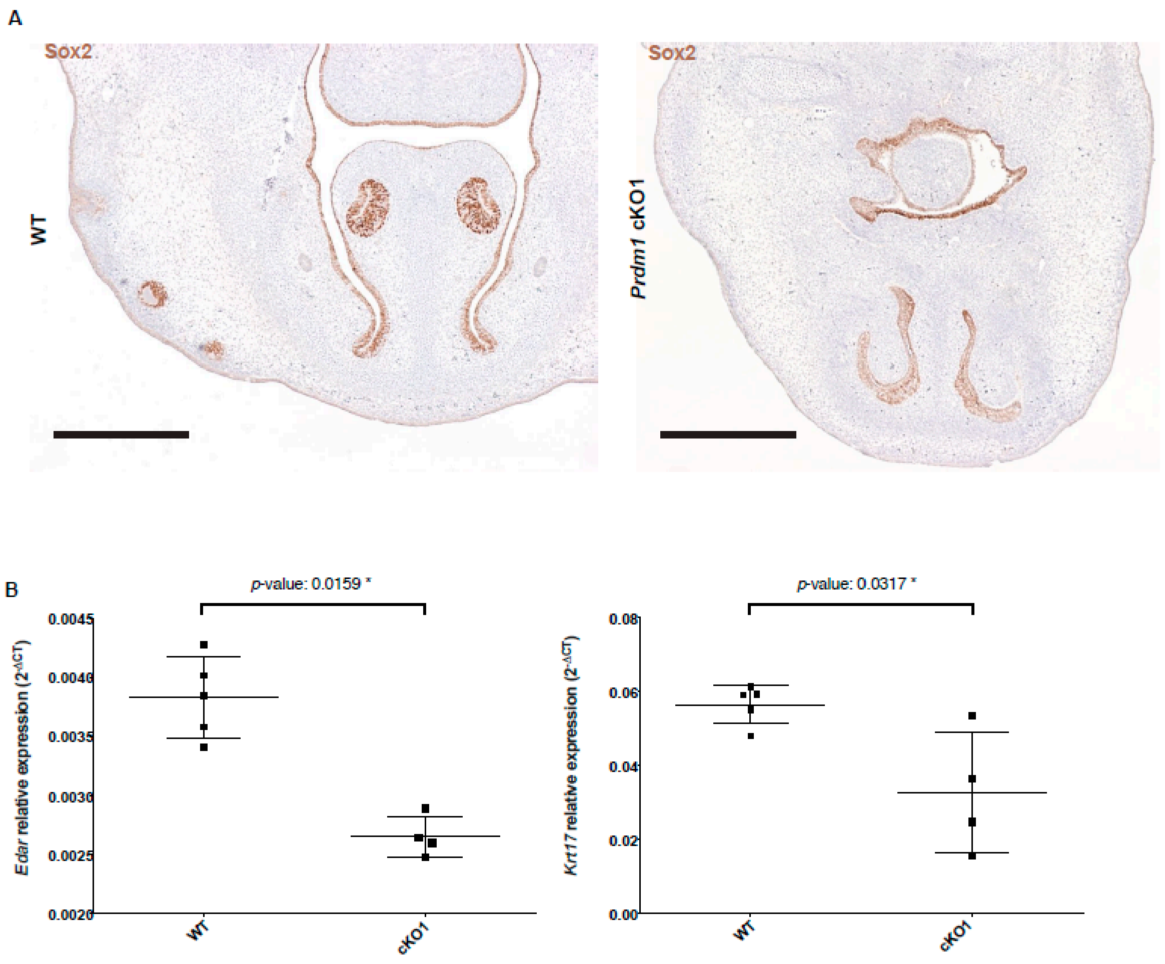

Figure S2. (A) Expression of Sox2 in wild-type and *Prdm1* cKO1 whisker pad at E13.5. No dermal condensate can be detected in cKO1 embryos. Scale bar: 500 µm (B) *Edar* and *Krt17* expression evaluated by RT-qPCR on E13 whisker pads of wild-type and cKO1 mice.

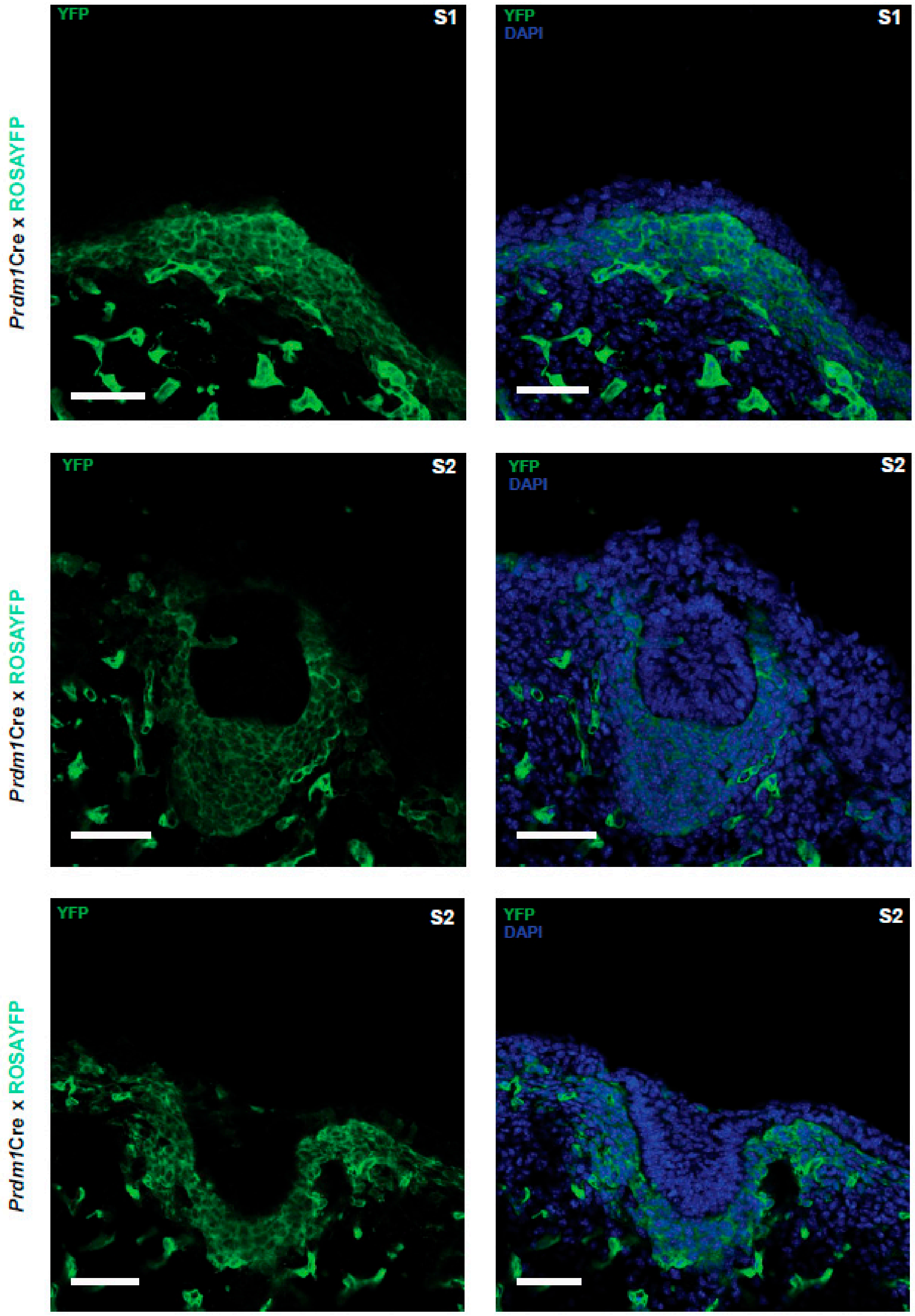

Figure S3. *Prdm1*Cre lineage tracing in developing whisker pads. *Prdm1*Cre was crossed with a ROSAYFP strain and developing whisker pads were analyzed at E12.5 and E13. YFP-positive cells encompass the developing whisker. Scale bar: 50 μm.

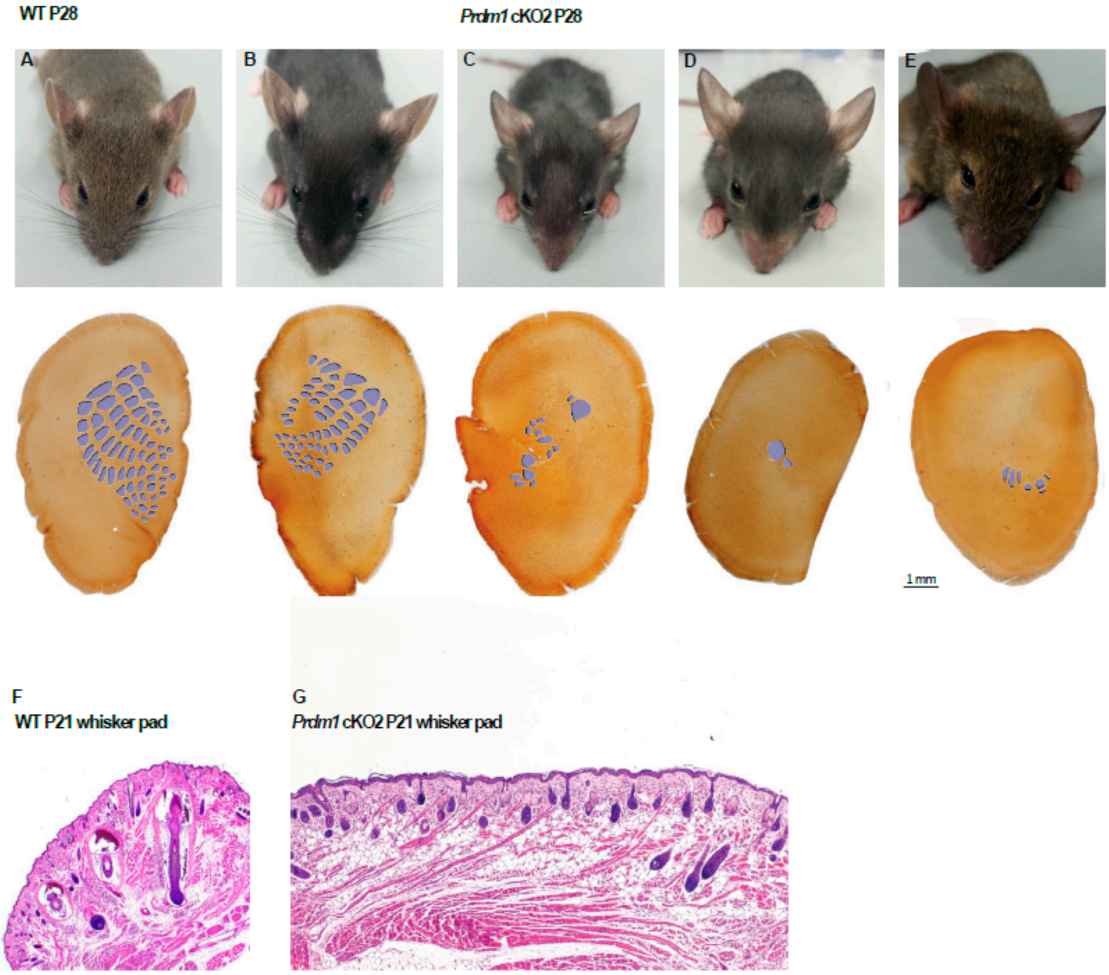

Figure S4. *Wnt1*Cre driven homozygous *Prdm1* knockout mice (cKO2) lack almost all the macro vibrissae except for the 1–3 distal ones of the first row, as observed both macroscopically and microscopically (C,D,E) compared to wild-type animals (A,B). The barrel cortices of the correspondent animals were stained with cytochrome oxidase and revealed a major rearrangement. Residual macro vibrissae are represented by enlarged barrels; the barrels representing micro vibrissae are still present, though highly disorganized. cKO2 whisker pads display unperturbed pelage hair follicle formation at P21 (G) compared to wild-type animals (F).

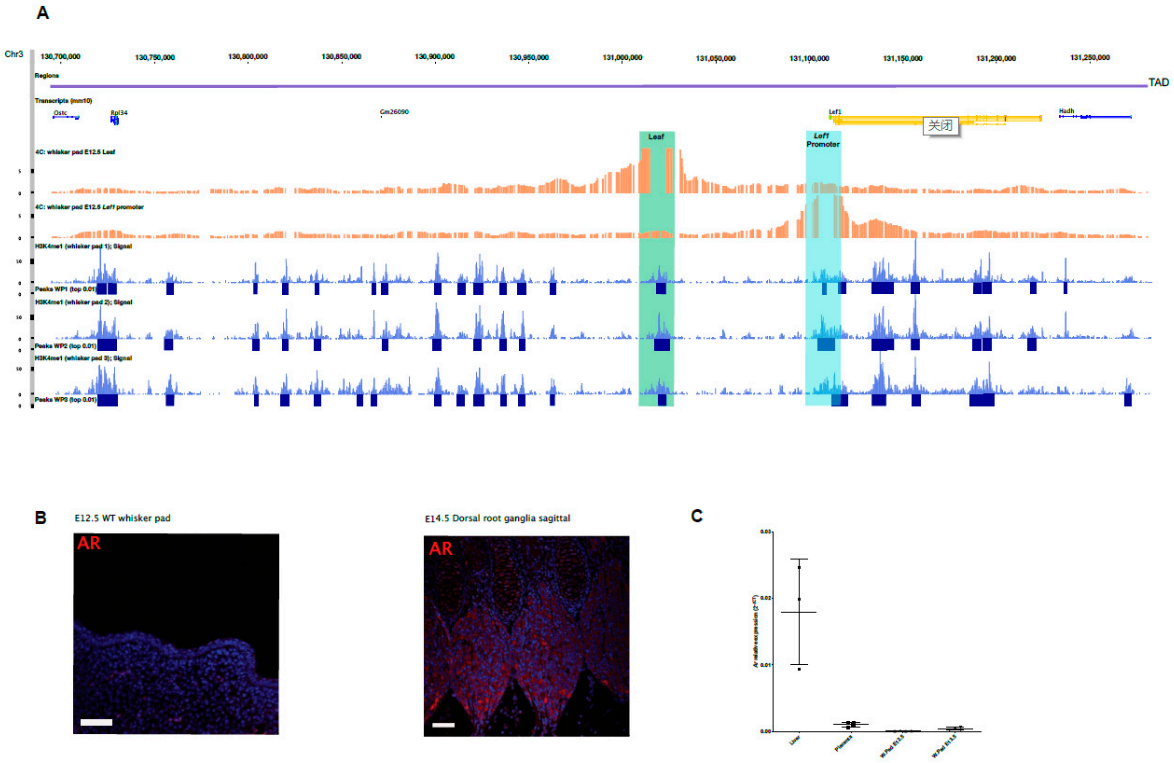

Figure S5. (A) Additional H3K4me1 profiles across all three CUT&Tag replicates. The green box indicates the genomic region where Leaf resides. (B) Ar IHC shows no expression in the E12.5 whisker pad compared to the dorsal root ganglia (positive control). (C)Validation of the lack of *Ar* expression in E12.5 whisker pad by RT-qPCR.

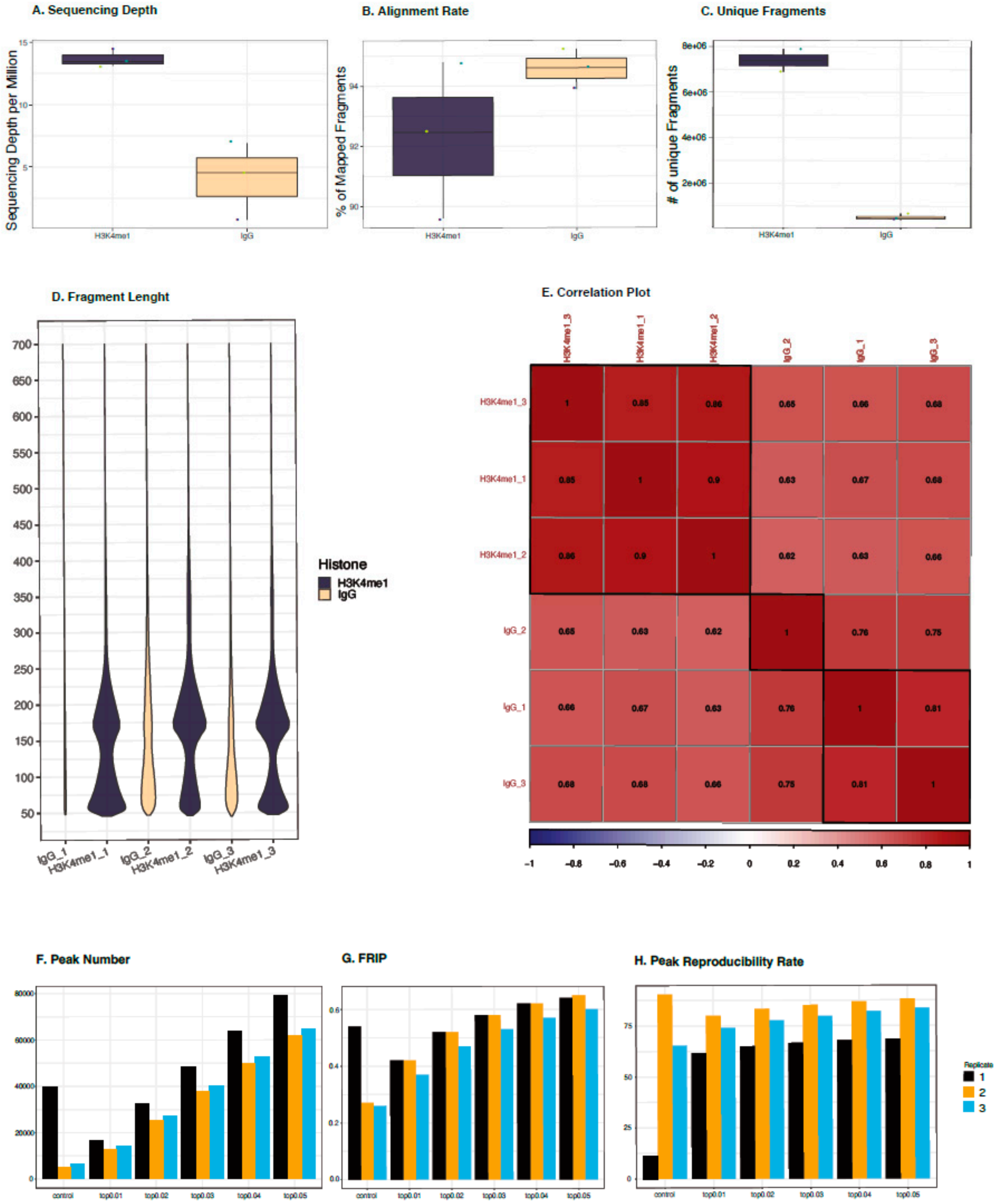

Figure S6. Quality controls of CUT&Tag H3K4me1 in E12 whisker pads. A. Sequencing depth B. Alignment Rate C. Unique Fragments D. Fragment Length E. Correlation plot between the H3k4me1 samples and the IgG controls. F. Number of significant H3K4me1 peaks with or without IgG control G. Fraction of Reads in Peaks in the different sets of peaks H. Peak reproducibility rate.
